# Cocainism

**Published:** 1899-11

**Authors:** J. Harrington Beynon


ARTICLE VII.

### COCAINISM.

J. HARRINGTON BEYNON, M. D.

Never from the frosty hills of the North, or the warm pregnant sod of the South, nor from the arid plains of the East or the virile soil of the West springs a shrub known alike to civilian and barbarian so fraught with possibilities for weal or woe to mankind as that veritable "bush of good or evil" Erythroxylon Coca. Poppy and Cannabis are harmless as milk in comparison.

No drug known to Materia Medica when taken habitually is followed in so short a time by such dire and calamitous results, such utter wreck and ruin, mental and physical as cocaine.

The average life of the cocaine debauchee is about two years, at the expiration of which he will die of insomnia or fall a ready victim to some intercurrent disease, if he escapes suicide or death from marasmus so long.

For centuries the coca plant has been known and used by the Indians of South America. The leaves, mixed with alkaline ash, are chewed for their sustaining and invigorating effect, banishing hunger, thirst and fatigue for days together. But even the native finally succumbs to its poisonous properties, losing appetite, flesh, vigor and ultimately his life from the marasmus which continued chewing induces.

Some fifty million pounds of coca leaves are annually harvested in Peru and Bolivia.

For the prevalence of the coca habit or cocainism in the United States and France, particularly in New York, Paris, New Orleans and other great centers, coca wines, coca lozenges, catarrh snuffs, sundry panaceas and cure-alls containing cocaine in dangerous amounts are largely, if not mainly, responsible.

The formation of this habit is most insidious, stealing upon one like a thief in the night.

Coca lozenges for chronic sore throat, catarrh snuffs for colds or a glass of coca wine for that tired feeling, frequently repeated, soon become a necessity, and long before the unsuspecting victim is himself aware of his bondage the line of safety is crossed and he stands fettered with chains requiring "iron bars and perspiration" to break.

The coca habit is more easily and readily acquired than that of opium, chloral or absinthe, because of the guileless form in which it is presented. It is many times harder to cure and decidedly more destructive. Most habitues who recover are those who take morphine in conjunction. The former drug seems to neutralize the effect, calms the raging nerves and holds the frenzied heart in check.

No sooner is the habit formed than disintegration begins; first the soft tissues, then the bony system, even the teeth become chalky and brittle and the enamel crumbles away.

The majority of victims seem offered upon the professional altar. Those whose vocations call for mental exhilaration and heightened imagination here find a ready but treacherous ally. The Magdalens of to-day and all those who wish to forget, herein also find surcease from sorrow.

The most deplorable cases are those resulting from the hypodermic use of cocaine solution, and in these the effect at first is one of pleasure; the eye is brightened, the color heightened, the expression animated, fatigue is banished, the imagination enriched and brilliant thoughts coin themselves into words. The conversation becomes witty and sparkling, dull care takes wings, and the troubles of the hour are remembered as pleasures that have passed.

While under its influence the victim becomes insensible to hunger, heat and cold. The chilling "mistral" or the scorching "simoon" is alike to him but a laughing zephyr.

Life is a perennial spring and its beaker seems filled to the brim. The day is bright, but the light is fleeting, fleeting, for the night cometh! A night cold and dark and pitiless, without a star, without a dawn! As the days pass more and more of the drug is needed to produce even transient stimulation—the dose to-day is doubled tomorrow.

Ultimately the appetite fails, the strength wanes, the muscles tremble, the face becomes pale and thin, and the mind enfeebled becomes a prey to hallucinations and delusions of persecution.

Degradation is now profound and we have "a gaunt, hollow-eyed spectre, whose demented functions move only in a pitiless circle of self intoxication—ghastly automatism," whose only joy is the prick of the hypodermic needle.

I have in mind five typical cases of cocainism which came under my personal observation. They were men of ability and promise, of mental and moral stamina above the average.

Two were doctors who took the drug for its own sake to relieve depression and fatigue. One of these severed his connection with the things of earth with pistol ball through the brain. The other died from an over-dose. The third, a divinity student, cut his throat with a penknife.

The fourth and fifth were men of affairs, who, under wise and sympathetic treatment, partially recovered, but to-day are suffering the slings and stings of outraged nature.

I recall a sixth case—would that I could forget it—of a journalist who in his moral abasement sunk so near the brink of the "pit" that the honor of his wife became a thing of barter and sale, for money to buy the accursed drug.

Does it not behoove us all to handle and prescribe this leaf-o'-the-hidden-thorn with the most careful intelligence?

Then let us be advised, for the habit once formed in the majority of cases proves fatal—like the "primrose path of dalliance," it leads to debility, insomnia, insanity and death. But follow rather the lead of kindly nature—"her ways are the ways of pleasantness and all her paths are peace," thus: When weary and worn, leave coca alone. Rest! Sleep!—*Alkaloidal Clinic.*

